# Data donation of individual shopping data to help predict the occurrence of disease: A pilot study linking individual loyalty card and health survey data to investigate COVID-19

**DOI:** 10.23889/ijpds.v8i3.2273

**Published:** 2023-09-11

**Authors:** Elizabeth Dolan, James Goulding, Anya Skatova

**Affiliations:** 1 N/LAB Nottingham University Business School, University of Nottingham; 2 Department of Population Health Sciences, Bristol Medical School, University of Bristol

## Introduction & Background

Previous studies have found shopping data could increase the predictive accuracy of disease surveillance systems and illuminate behavioural responses in the self-management of symptoms of disease. Yet, accessing individual sales datasets for linkage to health datasets is challenging, and the recruitment of appropriate sample sizes for medical research has been limited.

## Objectives & Approach

### Objectives

Collect and link individual health data to individual shopping data to investigate COVID-19. Assess the feasibility of scaling-up this method, and use the collected data to investigate using loyalty card data in machine learning (ML) models for disease.

### Methods

Based on recommendations on the public’s preferences for data donation a new protocol was designed for collecting, linking and analysing shopping and health data. Participants were requested to use the Tesco Clubcard website data portability function to share their loyalty card data and complete an online health survey. An exploratory data analysis was conducted on the linked dataset. Participants were recruited online (18/01/2022 to 04/02/2022) with a recruitment target of 200.

## Relevance to Digital Footprints

The collection and analysis of individual transactional sales data for health research.

## Results

197 participants shared their Tesco Clubcard and health survey data. Tesco Clubcard data contained 893,414 transactions of 65,310 uniquely named items purchased from 2015 to 2022. Average transactions per participant were 4,653 (SD 5256) and average timeframe recorded was five years 6 months and 30 days (SD 836 days). A total of 6,993 medication sales were recorded accounting for 1% of sales, 81% (159/197) of participants bought medications and the average was 44 (STD 68) medications per individual. Most participants (196/197) shared their health status in the survey, and 94% (81/86) of those on medication shared the medication names. Participants reported donating their data to do good (79%, 155/197), help the NHS (77%, 152/197), be socially responsible (74%, 144/197) and because data was secure and anonymised (78%, 153/197).

## Conclusions & Implications

Using this new protocol which enables convenient data sharing with transparent data safeguards, the public were willing to share both their shopping and health data for research into COVID-19. To apply robust ML analysis, particularly to explore self-medication at an individual level, recruitment must be significantly scaled to collect data from enough individuals with high sales and regular shopping frequency, or new ML techniques developed to address sparseness in loyalty card data of key purchasing events related to health. The study suggests public readiness to share shopping data for health research, but investment is needed for large-scale data collection and AI application.

